# Author Correction: Trimodal single-cell profiling reveals a novel pediatric CD8αα^+^ T cell subset and broad age-related molecular reprogramming across the T cell compartment

**DOI:** 10.1038/s41590-024-01757-5

**Published:** 2024-01-24

**Authors:** Zachary Thomson, Ziyuan He, Elliott Swanson, Katherine Henderson, Cole Phalen, Samir Rachid Zaim, Mark-Phillip Pebworth, Lauren Y. Okada, Alexander T. Heubeck, Charles R. Roll, Veronica Hernandez, Morgan Weiss, Palak C. Genge, Julian Reading, Josephine R. Giles, Sasikanth Manne, Jeanette Dougherty, C. J. Jasen, Allison R. Greenplate, Lynne A. Becker, Lucas T. Graybuck, Suhas V. Vasaikar, Gregory L. Szeto, Adam K. Savage, Cate Speake, Jane H. Buckner, Xiao-jun Li, Thomas F. Bumol, E.John Wherry, Troy R. Torgerson, Laura A. Vella, Sarah E. Henrickson, Peter J. Skene, Claire E. Gustafson

**Affiliations:** 1 Allen Institute for Immunology, Seattle, WA USA; 2 Department of Systems Pharmacology and Translational Therapeutics, University of Pennsylvania School of Medicine, Philadelphia, PA USA; 3 Immune Health, University of Pennsylvania Perelman School of Medicine, Philadelphia, PA USA; 4 Center for Interventional Immunology, Benaroya Research Institute at Virginia Mason, Seattle, WA USA; 5 Center for Translational Immunology, Benaroya Research Institute at Virginia Mason, Seattle, WA USA; 6 Institute for Immunology, University of Pennsylvania Perelman School of Medicine, Philadelphia, PA USA; 7 Department of Pediatrics, Children’s Hospital of Philadelphia and the University of Pennsylvania Perelman School of Medicine, Philadelphia, PA USA; 8 Present Address: Department of Genome Sciences, University of Washington School of Medicine, Seattle, WA USA; 9 Present Address: Microbiology, Immunology and Cancer Biology (MICaB) Program, University of Minnesota, Minneapolis, Minneapolis, MN USA; 10 Present Address: Seagen, Bothell, WA USA

**Keywords:** Translational immunology, Cellular immunity, Chromatin analysis, RNA sequencing, Next-generation sequencing

Correction to: *Nature Immunology* 10.1038/s41590-023-01641-8, published online 16 October 2023.

In the version of the article initially published, Figs. 1b, 4f and g, 5i and j, and Extended Data Fig. 3c contained errors caused by a labeling mistake in some donors in the scRNA-seq confirmatory cohort. Following reanalysis of the data, the figures have been replaced in the HTML and PDF versions of the article. The original and revised figures are shown in the Supplementary Information accompanying this amendment. There is no change to the scientific conclusions of the article.

Supplementary Information is available in the online version of this amendment.

### Supplementary information

Supplementary Information Original and revised Figs. 1b, 4f and g, 5i and j, and Extended Data Fig. 3c

