# Supplementary material for: Trimodal single-cell profiling reveals a novel pediatric CD8αα+ T cell subset and broad age-related molecular reprogramming across the T cell compartment
Source: Nat Immunol. 2023 Oct 16;24(11):1947–59. doi: 10.1038/s41590-023-01641-8 (PMC10602854; doi:10.1038/s41590-023-01641-8)
Supplement: Supplementary file 1 — Reporting Summary [file 41590_2023_1641_MOESM1_ESM.pdf]

nature portfolio

Corresponding author(s): Skene and Gustafson

Last updated by author(s): 08/21/2023

Reporting Summary

Nature Portfolio wishes to improve the reproducibility of the work that we publish. This form provides structure for consistency and transparency in reporting. For further information on Nature Portfolio policies, see our Editorial Policies and the Editorial Policy Checklist.

Statistics

For all statistical analyses, confirm that the following items are present in the figure legend, table legend, main text, or Methods section.

n/a	Confirmed	
☐	☒	The exact sample size (*n*) for each experimental group/condition, given as a discrete number and unit of measurement
☐	☒	A statement on whether measurements were taken from distinct samples or whether the same sample was measured repeatedly
☐	☒	The statistical test(s) used AND whether they are one- or two-sided<br>*Only common tests should be described solely by name; describe more complex techniques in the Methods section.*
☐	☒	A description of all covariates tested
☐	☒	A description of any assumptions or corrections, such as tests of normality and adjustment for multiple comparisons
☐	☒	A full description of the statistical parameters including central tendency (e.g. means) or other basic estimates (e.g. regression coefficient) AND variation (e.g. standard deviation) or associated estimates of uncertainty (e.g. confidence intervals)
☐	☒	For null hypothesis testing, the test statistic (e.g. *F*, *t*, *r*) with confidence intervals, effect sizes, degrees of freedom and *P* value noted<br>*Give P values as exact values whenever suitable.*
☒	☐	For Bayesian analysis, information on the choice of priors and Markov chain Monte Carlo settings
☒	☐	For hierarchical and complex designs, identification of the appropriate level for tests and full reporting of outcomes
☒	☐	Estimates of effect sizes (e.g. Cohen's *d*, Pearson's *r*), indicating how they were calculated

Our web collection on statistics for biologists contains articles on many of the points above.

Software and code

Policy information about availability of computer code

Data collection	Cytek SpectroFlo software (Version 2.0.2), BD FACSChorus (v2)
Data analysis	FlowJo (V10.8), Graphpad Prism 9 for macOS (v9.5.0), R 4.1.2, CellRanger 7.1, Cell Ranger Arc 1.0.1, Seurat 4.1.0, ggplot2 3.4.0, Matrix 1.4-0, rhdf5 2.38.0, H5weaver 1.2.0, dplyr 1.0.8, viridis 0.6.2, harmony 0.1.0, Nebulosa 1.4.0, GenomicRanges 1.46.1, SummarizedExperiment 1.24.0, ArchR 1.0.2, ggrepel 0.9.1, scales 1.2.1, tidyverse 1.3.1, tidyr 1.2.0, plyranges 1.14.0 TxDb.Hsapiens.USCS.hg38.refGene 3.13.0, org.Hs.eg.db 3.14.0, OrganismDbi 1.36.0, ggbio 1.42.0, caret 6.0-93, ggdist 3.2.0, gghalves 0.1.4, ggraph 2.1.0, clustree 0.5.0, ggpmisc 0.5.2, UpSetR 1.4.0, doParallel 1.0.17, stringr 1.4.0. Code used for analysis and figure generation in this manuscript is available on Github (https://github.com/aifimmunology/Aging_Tcell_TEA-seq).

For manuscripts utilizing custom algorithms or software that are central to the research but not yet described in published literature, software must be made available to editors and reviewers. We strongly encourage code deposition in a community repository (e.g. GitHub). See the Nature Portfolio guidelines for submitting code & software for further information.

Data

Policy information about availability of data

All manuscripts must include a data availability statement. This statement should provide the following information, where applicable:

- Accession codes, unique identifiers, or web links for publicly available datasets
- A description of any restrictions on data availability
- For clinical datasets or third party data, please ensure that the statement adheres to our policy

Raw data is deposited in the NCBI Database of Genotypes and Phenotypes (dbGaP, study ID: phs003400.v1) for controlled access. Processed data is deposited in the NCBI Gene Expression Omnibus database (GEO, Series Accession ID: GSE214546). External cord blood (ID: GSE157007) and pediatric MIS-C (ID: GSE166489) datasets are from the GEO database. Thymus dataset is from Array Express (accession #E-MTAB-8581). A custom collection of genesets that included the Hallmark v7.2 genesets, KEGG v7.2, and Reactome v7.2 from the Molecular Signatures Database (MSigDB, v4.0) was used as the pathway database in GSEA analyses.

Human research participants

Policy information about studies involving human research participants and Sex and Gender in Research.

Reporting on sex and gender	Our initial TEA-seq of 16 people studied only women, in order to reduce data variation in our cohort of 8 pediatric and 8 adult donors. However, in order to expand these data, we selected a followup cohort for scRNA-seq of 48 individuals with 24 female and 24 male subjects, with each age group equally distributed by sex. Fixed scRNAseq for stimulation was performed on 4 female pediatric donors from the TEA-seq cohort. The cohort demographics are provided in Supplemental Table 3.
Population characteristics	Healthy pediatric (11-13yr olds), young adults (25-35 yrs) and older adults (55-65 yrs) were recruited for these studies. Relevant covariates include age, sex and CMV infection status and are provided in Figure 1b and Supplemental Table 3.
Recruitment	For sequencing studies: Adult: Healthy 25-35 year old and 55-65 year old adult subjects were recruited from the greater Seattle area as part of the Sound Life project at Benaroya Research Institute (BRI). Patients were excluded from enrollment if they had a history of chronic disease, autoimmune disease, severe allergy, or chronic infection. Subjects enrolled by BRI were compensated for their time, effort and incidental expenses related to the research visits with $50 per research visit that involved a blood draw. Pediatric: Healthy 11-13 year old pediatric subjects were recruited from the greater Philadelphia area. Patients were excluded from enrollment if they had a history of immune deficiency, fever or antibiotic usage within the month prior to sample collection, chronic medication usage or BMI more than 2 standard deviations above or below the mean for their age. Pediatric subjects enrolled were compensated for their time and effort. Payments were structured to increase as the longitudinal visits progressed and ranged from $25 to $100 per research visit that involved a blood draw. In addition, small thank-you token gifts were available for the pediatric subjects to choose from after each visit.
Ethics oversight	All studies were approved by Institutional Review Boards at Benaroya Research Institute (adult cohorts), University of Pennsylvania (pediatric cohort) and/or Allen Institute (all sample usage). All adult participants gave informed consent prior to participation in these studies. Informed consent for participation of minors was obtained from a legally authorized representative of the child. If capable, the participating child also provided assent to participate in the study. Cord and peripheral blood samples for follow-up studies were purchased from Bloodworks Northwest (Seattle, WA) and BioIVT (Hicksville, NY) obtained with written informed consent and use approved by Allen Institute IRB.

Note that full information on the approval of the study protocol must also be provided in the manuscript.

Field-specific reporting

Please select the one below that is the best fit for your research. If you are not sure, read the appropriate sections before making your selection.

☒ Life sciences ☐ Behavioural & social sciences ☐ Ecological, evolutionary & environmental sciences

For a reference copy of the document with all sections, see nature.com/documents/nr-reporting-summary-flat.pdf

Life sciences study design

All studies must disclose on these points even when the disclosure is negative.

Sample size	TEAseq experiments included 16 donors (8 per age group), cohort scRNASeq experiments included 48 donors (16 per age group) and stimulation scRNAseq included 4 donors (pediatric only). Sample size power calculation was performed for our larger validation cohort. The minimum sample size required to identify a 1% change while controlling for Type I and Type II errors at =0.05, = 0.2, respectively, and applying an estimated frequencies standard deviation of =0.45, is n=5 per group for a two-sample t-test. Applying a sample size correction based on the Asymptotic Relative Efficiency (ARE) of the Mann Whitney U test (i.e., 15.7%) results in a minimum required sample size of n=6 per group to identify 1% differences to attain 80% power and control for Type I and II error rates at =0.05, =0.2, respectively. Thus we exceed the minimum required n=6 per age group.
Data exclusions	No data were excluded from analyses.

Replication	All experiments have been biologically replicated in at least three donors and results were successfully reproduced. All sequencing was performed using a common PBMC batch control for technical replication confirmation and normalization.
Randomization	For the TEA-seq dataset, randomization of the groups was not possible since the study design was to compare donors based on specific clinical parameters; age group, and when appropriate, CMV infection status. However, sample were randomly distributed between batches of TEA-seq runs to mitigate assay variability. Samples used in scRNA-seq experiments were randomized across batches. Stimulation scRNA -seq experiments were performed as a single batch.
Blinding	Experiments and analyses were not performed blinded as the same investigator(s) oversaw the sample processing, data generation and data analyses.

Reporting for specific materials, systems and methods

We require information from authors about some types of materials, experimental systems and methods used in many studies. Here, indicate whether each material, system or method listed is relevant to your study. If you are not sure if a list item applies to your research, read the appropriate section before selecting a response.

Materials & experimental systems

n/a	Involved in the study
☐	☒ Antibodies
☒	☐ Eukaryotic cell lines
☒	☐ Palaeontology and archaeology
☒	☐ Animals and other organisms
☒	☐ Clinical data
☒	☐ Dual use research of concern

Methods

n/a	Involved in the study
☒	☐ ChIP-seq
☐	☒ Flow cytometry
☒	☐ MRI-based neuroimaging

Antibodies

Antibodies used

TEA-seq panel
Antibody TotalSeq™-A0151 anti-human CD152 (CTLA-4) Antibody BNI3 BioLegend Cat# 369619, RRID:AB_2734423 (0.175 µg per million cells)
Antibody TotalSeq™-A0071 anti-human CD194 (CCR4) Antibody L291H4 BioLegend Cat# 359423, RRID:AB_2749979 (0.175 µg per million cells)
Antibody TotalSeq™-A0143 anti-human CD196 (CCR6) Antibody G034E3 BioLegend Cat# 353437, RRID:AB_2750534 (0.175 µg per million cells)
Antibody TotalSeq™-A0189 anti-human CD244 (2B4) Antibody C1.7 BioLegend Cat# 329527, RRID:AB_2750007 (0.175 µg per million cells)
Antibody TotalSeq™-A0396 anti-human CD26 Antibody BA5b BioLegend Cat# 302720, RRID:AB_2734261 (0.175 µg per million cells)
Antibody TotalSeq™-A0102 anti-human CD294 (CRTH2) Antibody BM16 BioLegend Cat# 350127, RRID:AB_2734360 (0.2 µg per million cells)
Antibody TotalSeq™-A0576 anti-human CD49d Antibody 9F10 BioLegend Cat# 304337, RRID:AB_2783166 (0.175 µg per million cells)
Antibody TotalSeq™-A0171 anti-human/mouse/rat CD278 (ICOS) Antibody C398.4A BioLegend Cat# 313555, RRID:AB_2800824 (0.05 µg per million cells)
Antibody TotalSeq™-A0161 anti-human CD11b Antibody ICRF44 BioLegend Cat# 301353, RRID:AB_2734249 (0.05 µg per million cells)
Antibody TotalSeq™-A0053 anti-human CD11c Antibody S-HCL-3 BioLegend Cat# 371519, RRID:AB_2749971 (0.025 µg per million cells)
Antibody TotalSeq™-A0390 anti-human CD127 (IL-7Rα) Antibody A019D5 BioLegend Cat# 351352, RRID:AB_2734366 (0.075 µg per million cells)
Antibody TotalSeq™-A0083 anti-human CD16 Antibody 3G8 BioLegend Cat# 302061, RRID:AB_2734255 (0.05 µg per million cells)
Antibody TotalSeq™-A0408 anti-human CD172a (SIRPα) Antibody 15-414 BioLegend Cat# 372109, RRID:AB_2783285 (0.25 µg per million cells)
Antibody TotalSeq™-A0144 anti-human CD185 (CXCR5) Antibody J252D4 BioLegend Cat# 356937, RRID:AB_2750356 (0.125 µg per million cells)
Antibody TotalSeq™-A0181 anti-human CD21 Antibody Bu32 BioLegend Cat# 354915, RRID:AB_2750006 (0.05 µg per million cells)
Antibody TotalSeq™-A0085 anti-human CD25 Antibody BC96 BioLegend Cat# 302643, RRID:AB_2734258 (0.08 µg per million cells)
Antibody TotalSeq™-A0154 anti-human CD27 Antibody O323 BioLegend Cat# 302847, RRID:AB_2750000 (0.05 µg per million cells)
Antibody TotalSeq™-A0088 anti-human CD279 (PD-1) Antibody EH12.2H7 BioLegend Cat# 329955, RRID:AB_2734322 (0.2 µg per million cells)
Antibody TotalSeq™-A0406 anti-human CD304 (Neuropilin-1) Antibody 12C2 BioLegend Cat# 354525, RRID:AB_2783261 (0.05 µg per million cells)
Antibody TotalSeq™-A0410 anti-human CD38 Antibody HB-7 BioLegend Cat# 356635, RRID:AB_2800967 (0.05 µg per million cells)
Antibody TotalSeq™-A0176 anti-human CD39 Antibody A1 BioLegend Cat# 328233, RRID:AB_2750005 (0.075 µg per million cells)
Antibody TotalSeq™-A0072 anti-human CD4 Antibody RPA-T4 BioLegend Cat# 300563, RRID:AB_2734247 (0.1 µg per million cells)
Antibody TotalSeq™-A0047 anti-human CD56 (NCAM) Antibody 5.1H11 BioLegend Cat# 362557, RRID:AB_2749970 (0.1 µg per million cells)
Antibody TotalSeq™-A0394 anti-human CD71 Antibody CY1G4 BioLegend Cat# 334123, RRID:AB_2800884 (0.05 µg per million cells)
Antibody TotalSeq™-A0080 anti-human CD8a Antibody RPA-T8 BioLegend Cat# 301067, RRID:AB_2734248 (0.2 µg per million cells)
Antibody TotalSeq™-A0006 anti-human CD86 Antibody IT2.2 BioLegend Cat# 305443, RRID:AB_2734273 (0.05 µg per million cells)

Antibody TotalSeq™-A0581 anti-human TCR Vα7.2 Antibody 3C10 BioLegend Cat# 351733, RRID:AB_2783246 (0.0625 µg per million cells)
Antibody TotalSeq™-A0145 anti-human CD103 (Integrin αE) Antibody Ber-ACT8 BioLegend Cat# 350231, RRID:AB_2749996 (0.2 µg per million cells)
Antibody TotalSeq™-A0168 anti-human CD57 Recombinant Antibody QA17A04 BioLegend Cat# 393319, RRID:AB_2810588 (0.2 µg per million cells)
Antibody TotalSeq™-A0146 anti-human CD69 Antibody FN50 BioLegend Cat# 310947, RRID:AB_2749997 (0.2 µg per million cells)
Antibody TotalSeq™-A0242 anti-human CD192 (CCR2) Antibody K036C2 BioLegend Cat# 357229, RRID:AB_2750501 (0.25 µg per million cells)
Antibody TotalSeq™-A0063 anti-human CD45RA Antibody HI100 BioLegend Cat# 304157, RRID:AB_2734267 (0.25 µg per million cells)
Antibody TotalSeq™-A0156 anti-human CD95 (Fas) Antibody DX2 BioLegend Cat# 305649, RRID:AB_2750368 (0.25 µg per million cells)
Antibody TotalSeq™-A0159 anti-human HLA-DR Antibody L243 BioLegend Cat# 307659, RRID:AB_2750001 (0.05 µg per million cells)
Antibody TotalSeq™-A0153 anti-human KLRG1 (MAFA) Antibody SA231A2 BioLegend Cat# 367721, RRID:AB_2750373 (0.25 µg per million cells)
Antibody TotalSeq™-A0355 anti-human CD137 (4-1BB) Antibody 4B4-1 BioLegend Cat# 309835, RRID:AB_2783173 (0.25 µg per million cells)
Antibody TotalSeq™-A0149 anti-human CD161 Antibody HP-3G10 BioLegend Cat# 339945, RRID:AB_2749998 (0.1 µg per million cells)
Antibody TotalSeq™-A0140 anti-human CD183 (CXCR3) Antibody G025H7 BioLegend Cat# 353745, RRID:AB_2749993 (0.25 µg per million cells)
Antibody TotalSeq™-A0896 anti-human CD85j (ILT2) Antibody GHI/75 BioLegend Cat# 333723, RRID:AB_2814225 (0.1 µg per million cells)
Antibody TotalSeq™-A0179 anti-human CX3CR1 Antibody K0124E1 BioLegend Cat# 355709, RRID:AB_2832698 (0.1 µg per million cells)
Antibody TotalSeq™-A0169 anti-human CD366 (Tim-3) Antibody F38-2E2 BioLegend Cat# 345047, RRID:AB_2800924 (0.2 µg per million cells)
Antibody TotalSeq™-A0005 anti-human CD80 Antibody 2D10 BioLegend Cat# 305239, RRID:AB_2749958 (0.25 µg per million cells)
Antibody TotalSeq™-A0148 anti-human CD197 (CCR7) Antibody G043H7 BioLegend Cat# 353247, RRID:AB_2750357 (0.5 µg per million cells)
Antibody TotalSeq™-A0386 anti-human CD28 Antibody CD28.2 BioLegend Cat# 302955, RRID:AB_2783159 (0.5 µg per million cells)
Antibody TotalSeq™-A0031 anti-human CD40 Antibody 5C3 BioLegend Cat# 334346, RRID:AB_2749968 (0.375 µg per million cells)
Antibody TotalSeq™-A0087 anti-human CD45RO Antibody UCHL1 BioLegend Cat# 304255, RRID:AB_2734268 (0.5 µg per million cells)
Antibody TotalSeq™-A0224 anti-human TCR α/β Antibody IP26 BioLegend Cat# 306737, RRID:AB_2783167 (0.375 µg per million cells)
Antibody TotalSeq™-A0139 anti-human TCR γ/δ Antibody B1 BioLegend Cat# 331229, RRID:AB_2734325 (0.25 µg per million cells)
Antibody TotalSeq™-A0089 anti-human TIGIT (VSTM3) Antibody A15153G BioLegend Cat# 372725, RRID:AB_2734426 (0.5 µg per million cells)
Antibody TotalSeq™-A0158 anti-human CD134 (OX40) Antibody Ber-ACT35 BioLegend Cat# 350033, RRID:AB_2783245 (0.5 µg per million cells)
Antibody TotalSeq™-A0032 anti-human CD154 Antibody 24-31 BioLegend Cat# 310843, RRID:AB_2734283 (0.5 µg per million cells)
Antibody TotalSeq™-A0584 anti-human TCR Vα24-Jα18 (iNKT cell) Antibody 6B11 BioLegend Cat# 342923, RRID:AB_2783227 (0.5 µg per million cells)
Antibody TotalSeq™-A0180 anti-human CD24 Antibody ML5 BioLegend Cat# 311137, RRID:AB_2750374 (0.5 µg per million cells)
Antibody TotalSeq™-A0830 anti-human CD319 (CRACC) Antibody 162.1 BioLegend Cat# 331821, RRID:AB_2800872 (0.5 µg per million cells)
Antibody TotalSeq™-A0090 Mouse IgG1, κ isotype Ctrl Antibody MOPC-21 BioLegend Cat# 400199, RRID:AB_2868412 (0.5 µg per million cells)
Flow PBMC phenotyping panel
Antibody Mouse anti-human CD3/BUV395 UCHT1 BD Bioscience Cat# 563546, RRID:AB_2744387 2uL per sample
Antibody Mouse anti-human CD45/BUV496 HI30 BD Bioscience Cat# 624283 2uL per sample
Antibody Mouse anti-human CD8/BUV737 RPA-T8 BD Bioscience Cat# 624286 0.5uL per sample
Antibody Mouse anti-human CD127/BV711 A019D5 BioLegend Cat# 351328, RRID:AB_2562908 2uL per sample
Antibody Mouse anti-human CD197/PE-Cy7 G043H7 BioLegend Cat# 353226, RRID:AB_11126145 3uL per sample
Antibody Mouse anti-human CD14/BB660 MφP9 BD Bioscience Cat# 624295 0.5uL per sample
Antibody Mouse anti-human CD56/BUV563 NCAM16.2 BD Bioscience Cat# 612928 0.5uL per sample
Antibody Mouse anti-human CD19/BUV615 HIB19 BD Bioscience Cat# 624297 1uL per sample
Antibody Mouse anti-human CD27/BUV661 L128 BD Bioscience Cat# 624285 0.5uL per sample
Antibody Mouse anti-human CD39/BUV805 Tu66 BD Bioscience Cat# 624287 1uL per sample
Antibody Mouse anti-human CD103/BV421 Ber-ACT8 BioLegend Cat# 350214, RRID:AB_2563514 1uL per sample
Antibody Mouse anti-human abTCR/BV480 IP26 BD Bioscience Cat# 624278 3uL per sample
Antibody Mouse anti-human CD223/BV605 11C3C65 BioLegend Cat# 369324, RRID:AB_2721541 2uL per sample
Antibody Mouse anti-human CD95/BV650 DX2 BioLegend Cat# 305642, RRID:AB_2632622 1uL per sample
Antibody Mouse anti-human CD278/BV750 DX29 BD Bioscience Cat# 624380 2uL per sample
Antibody Mouse anti-human CD45RA/BV786 HI100 BioLegend Cat# 304140, RRID:AB_2563816 1uL per sample
Antibody Mouse anti-human CD185/BB515 RF8B2 BD Bioscience Cat# 564624, RRID:AB_2738871 2uL per sample
Antibody Mouse anti-human CD4/BB700 SK3 BD Bioscience Cat# 566392, RRID:AB_2744421 2uL per sample
Antibody Mouse anti-human HLA-DR/BB790 G46-6 BD Bioscience Cat# 624296 1uL per sample
Antibody Mouse anti-human CD279/PE EH12.2H7 BioLegend Cat# 329906, RRID:AB_940483 2uL per sample
Antibody Mouse anti-human TIGIT/PE-Dazzle594 A15153G BioLegend Cat# 372716, RRID:AB_2632931 4uL per sample
Antibody Mouse anti-human CD38/PE-Cy5 HIT2 BD Bioscience Cat# 555461, RRID:AB_395854 5uL per sample
Antibody Mouse anti-human CD69/APC FN50 BioLegend Cat# 310910, RRID:AB_314845 2uL per sample
Antibody Mouse anti-human CD25/APC-R700 2A3 BD Bioscience Cat# 565106, RRID:AB_2744339 2uL per sample
Antibody Mouse anti-human KLRG1/APC-Fire750 SA231A2 BioLegend Cat# 367718, RRID:AB_2687392 1uL per sample
Flow sorting panel (Naïve CD4 T cells)
Antibody Brilliant Violet 421™ anti-human CD95 (Fas) Antibody DX2 BioLegend Cat# 305624, RRID:AB_2561830 2.5uL per sample
Antibody FITC anti-human CD3 Antibody UCHT1 BioLegend Cat# 300406, RRID:AB_314060 0.5uL per sample

Antibody PerCP/Cyanine5.5 anti-human CD27 Antibody O323 BioLegend Cat# 302820, RRID:AB_2073318 2uL per sample
Antibody PE anti-human CD197 (CCR7) Antibody G043H7 BioLegend Cat# 353204 2uL per sample
Antibody PE-Cy™7 Mouse Anti-Human CD4 Antibody SK3 BD Bioscience Cat# 557852, RRID:AB_396897 2uL per sample
Antibody APC anti-human CD45RA Antibody HI100 BioLegend Cat# 304112, RRID:AB_314416 2uL per sample
Antibody APC/Cyanine7 anti-human CD8a Antibody RPA-T8 BioLegend Cat# 301016, RRID:AB_314134 2uL per sample
Flow sorting panel (Total T cells for TEA-seq)
Antibody PE anti-human CD3 Antibody UCHT1 BioLegend Cat# 300441, RRID:AB_2562047 1uL per sample
Antibody FITC anti-human CD45 Antibody HI30 BioLegend Cat# 304038, RRID:AB_2562050 1uL per sample
Flow sorting panel (MNP-2 population)
Antibody FITC anti-human CD3 Antibody UCHT1 BioLegend Cat# 300406, RRID:AB_2562047 0.5uL per sample
Antibody PE-Cy™7 Mouse Anti-Human CD4 Antibody SK3 BD Bioscience Cat# 557852, RRID:AB_396897 2uL per sample
Antibody APC/Cyanine7 anti-human CD8a Antibody RPA-T8 BioLegend Cat# 301016, RRID:AB_314134 2uL per sample
Antibody APC anti-human CD45RA Antibody HI100 BioLegend Cat# 304112, RRID:AB_314416 2uL per sample
Antibody BV786 anti-human abTCR Antibody IP26 BD Bioscience Cat# 306742, RRID:AB_2783171 2uL per sample
Antibody BV421 anti-human CD244 (2B4) Antibody C1.7 BioLegend Cat# 329532, RRID:AB_2814194 2.5uL per sample
Antibody PE anti-human CD11b Antibody ICRF44 BD Bioscience Cat# 555388, RRID:AB_395789 5uL per sample
Flow MNP-2 phenotyping panel
Antibody AF488 anti-human CD45RA HI100 BioLegend Cat# 304114, RRID:AB_528816 1.25uL per sample
Antibody Spark Blue 550 anti-human CD8a SK1 BioLegend Cat# 344760, RRID:AB_2819983 0.6uL per sample
Antibody BV650 anti-human CD4 SK3 BD Bioscience Cat# 563875, RRID:AB_2744425 0.6uL per sample
Antibody BV750 anti-human TCR alpha-beta IP26 BioLegend Cat# 306746, RRID:AB_2810463 2.5uL per sample
Antibody BV480 anti-human CD19 HIB19 BD Bioscience Cat# 746457, RRID:AB_2743759 0.6uL per sample
Antibody PE-Cy7 anti-human CD197 (CCR7) G043H7 BioLegend Cat# 353226, RRID:AB_11126145 2.5uL per sample
Antibody BV421 anti-human CD95 (Fas) DX2 BioLegend Cat# 305624, RRID:AB_2561830 2.5uL per sample
Antibody BUV395 anti-human CD27 L128 BD Bioscience Cat# 563815, RRID:AB_2744349 1.25uL per sample
Antibody BUV805 anti-human CD3 UCHT1 BD Bioscience Cat# 612895, RRID:AB_2870183 2.5uL per sample
Antibody BUV496 anti-human CD45 HI30 BD Bioscience Cat# 750179, RRID:AB_2868405 0.6uL per sample
Antibody PE-Dazzle594 anti-human CD244 (2B4) C1.7 BioLegend Cat# 329521, RRID:AB_2572018 2.5uL per sample
Antibody BUV661 anti-human CD11b ICRF44 BD Bioscience Cat# 741601, RRID:AB_2916939 5uL per sample
Antibody BV480 anti-human CD14 MOP9 BD Bioscience Cat# 566141, RRID:AB_2739539 0.6uL per sample
Antibody PE anti-human CD360 (IL-21R 17A12 BioLegend Cat# 359505, RRID:AB_2562368 5uL per sample
Antibody APC anti-human CD8b QA20A40 BioLegend Cat# 376705, RRID:AB_2910430 5uL per sample
Flow CD8 activation panel
Antibody BUV395 anti-human CD71 M-A712 BD Bioscience Cat# 568523, RRID:AB_2937039 2uL per sample
Antibody BUV496 anti-human CD69 FN50 BD Bioscience Cat# 750214, RRID:AB_2874415 2uL per sample
Antibody BUV805 anti-human CD3 UCHT1 BD Bioscience Cat# 612895, RRID:AB_2870184 1uL per sample
Antibody BV421 anti-human CD25 BC96 Biolegend Cat# 302630, RRID:AB_11126749 2uL per sample
Antibody BV480 anti-human CD19 HIB19 BD Bioscience Cat# 746457, RRID:AB_2743759 1uL per sample
Antibody BV480 anti-human CD14 MOP9 BD Bioscience Cat# 566141, RRID:AB_2739539 1uL per sample
Antibody BV650 anti-human CD4 SK3 BD Bioscience Cat# 563875, RRID:AB_2744425 1uL per sample
Antibody BV750 anti-human TCR αß IP26 Biolegend Cat# 306746, RRID:AB_2810463 1uL per sample
Antibody AF488 anti-human CD45RA HI100 Biolegend Cat# 304114, RRID:AB_528816 1uL per sample
Antibody PerCP-Cy5.5 anti-human CD11b M1/70 Biolegend Cat# 101228, RRID:AB_893232 5uL per sample
Antibody PE anti-human CD360 (IL-21R) 17A12 Biolegend Cat# 359506, RRID:AB_2562369 5uL per sample
Antibody PE-Dazzle594 anti-human CD244 (2B4) C1.7 Biolegend Cat# 329521, RRID:AB_2572018 2uL per sample
Antibody PE-Cy7 anti-human CD197 (CCR7) G043H7 Biolegend Cat# 353226, RRID:AB_11126145 2uL per sample
Antibody APC anti-human CD8b QA20A40 Biolegend Cat# 376706, RRID:AB_2937040 1uL per sample
Antibody APC-Cy7 anti-human CD8a RPA-T8 Biolegend Cat# 301016, RRID:AB_314134 1uL per sample

Validation

All antibodies were purchased from established vendors with strict quality control assurances and validation statements can be found on the manufacturers' websites using the catalogue number or in the Antibody Registry database (https://antibodyregistry.org) via the provided RRID. TotalSeq antibodies were additionally titrated for optimal performance, with optimal concentrations listed above and in Supp Table 4.

Flow Cytometry

Plots

Confirm that:

☒ The axis labels state the marker and fluorochrome used (e.g. CD4-FITC).

☒ The axis scales are clearly visible. Include numbers along axes only for bottom left plot of group (a 'group' is an analysis of identical markers).

☒ All plots are contour plots with outliers or pseudocolor plots.

☒ A numerical value for number of cells or percentage (with statistics) is provided.

Methodology

Sample preparation

All blood samples were collected, processed to PBMCs using a Ficoll-based approach and frozen in FBS with 10% DMSO within 4 hours of blood draw. For follow-up studies, T cells were directly isolated from whole blood using RosettaSep Human T-cell Enrichment Cocktail then immediately cryopreserved as described above.

Instrument

Cytek Aurora (5 laser), BD Melody

Software	FlowJo v10.8, BD FACSChorus (v2), Cytek SpectroFlo software (Version 2.0.2)
Cell population abundance	The purity of sorted cell populations (true naive CD4 T cells, MNP-2, naive CD8 T cells) was greater than 88%.
Gating strategy	FSC-A/SSC-A was used to identify lymphocytes. FSC-H/FCS-W and SSC-H/SSC-W were used to remove doublets. Viability was determined using live/Dead stain, gating on negative (i.e., live) cells. T cells were determined by the presence of CD3 and the absence of CD19 and CD14. CD4, CD8, CD27, CD45RA, CCR7, CD127 and CD25 were used to determine T cell subsets. For naive CD4 follow-up studies, CD95 was additionally used to separate true naive T cells (CD95-negative). MNP-2 cells were determined by CD244 and CD11b co-expression within CD8+ TCRab+ T cells.

☒ Tick this box to confirm that a figure exemplifying the gating strategy is provided in the Supplementary Information.
