# Supplementary material for: Author Correction: Trimodal single-cell profiling reveals a novel pediatric CD8αα+ T cell subset and broad age-related molecular reprogramming across the T cell compartment
Source: Nat Immunol. 2024 Jan 24;25(3):577. doi: 10.1038/s41590-024-01757-5 (PMC10907283; doi:10.1038/s41590-024-01757-5)

Author Correction: Trimodal single-cell profiling reveals a novel pediatric CD8αα^{+} T cell subset and broad age-related molecular reprogramming across the T cell compartment

In the format provided by the authors and unedited

Original and revised Fig. 1b

Original

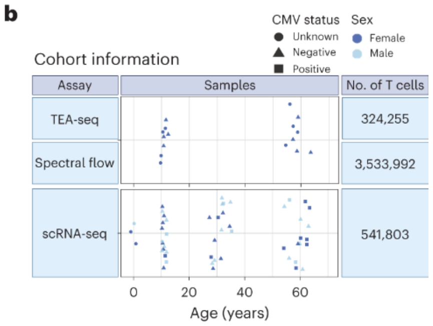

Corrected

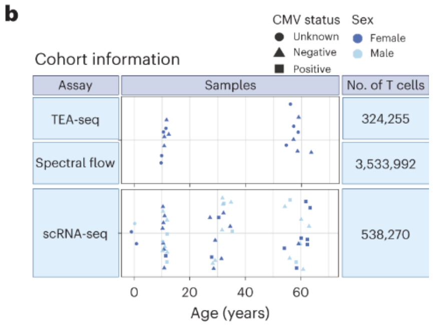

Original and revised Fig. 4f,g

Original

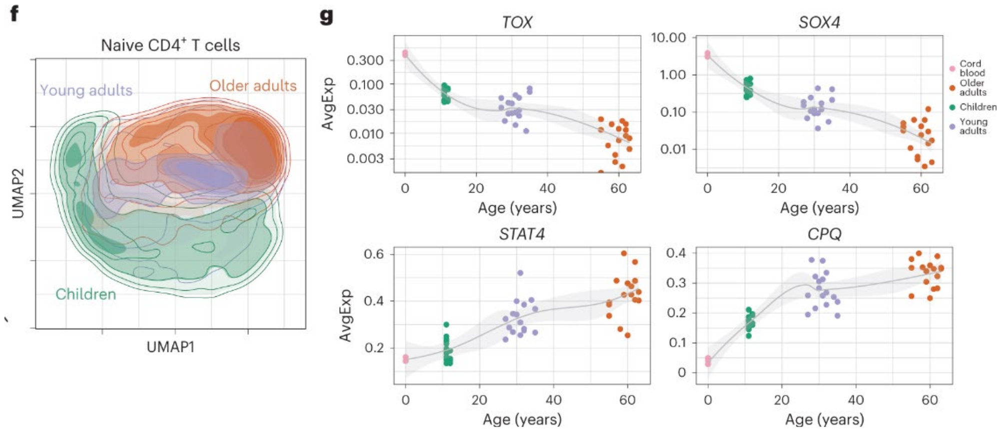

Corrected

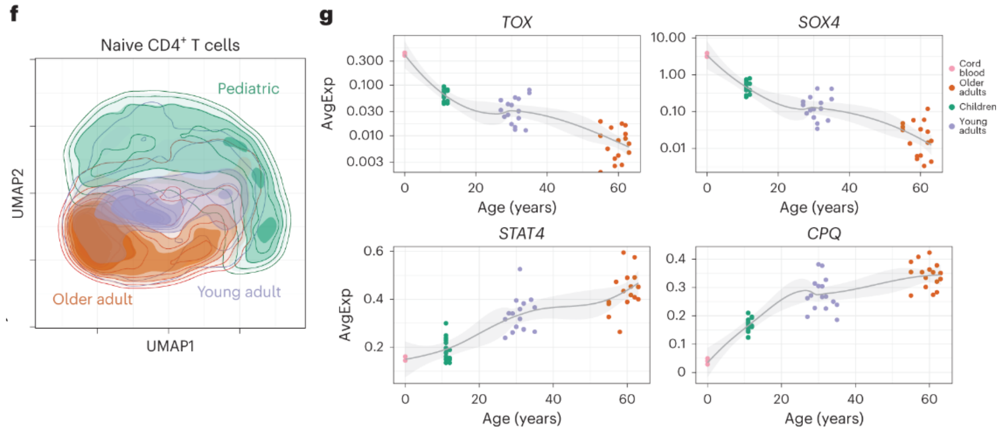

Original and revised Fig. 5i,j

Original

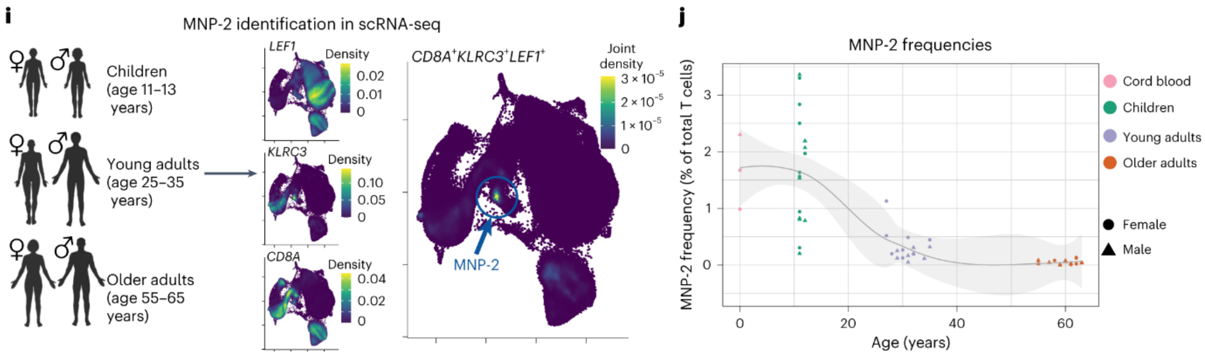

Corrected

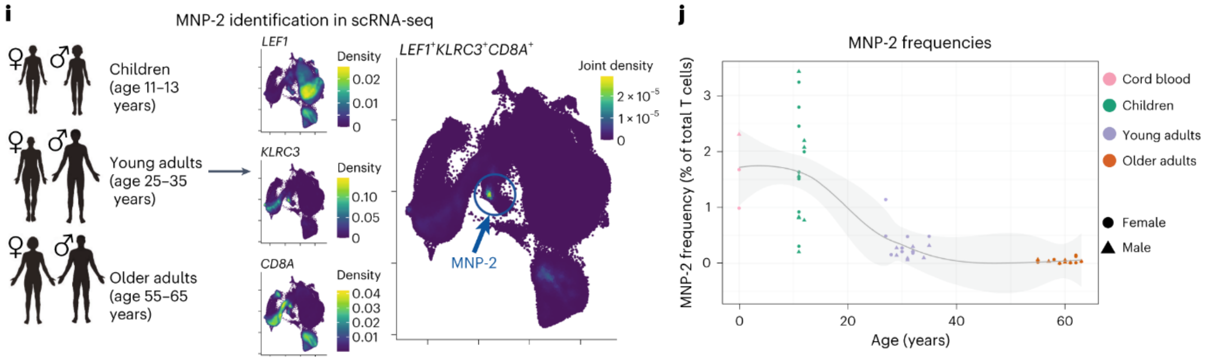

Original and revised Extended Data Fig. 3c

Original

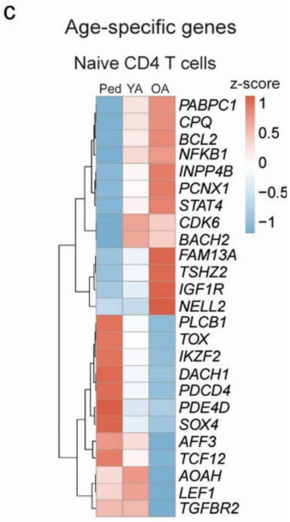

Corrected

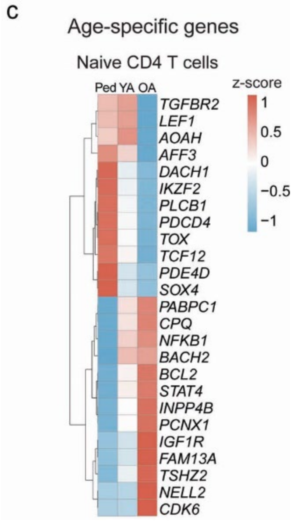

Supplement: Supplementary file 1 — Original and revised Figs. 1b, 4f and g, 5i and j, and Extended Data Fig. 3c [file 41590_2024_1757_MOESM1_ESM.pdf]
